# Acute Ischemic Stroke With Mild Symptoms–To Thrombolyse or Not to Thrombolyse?

**DOI:** 10.3389/fneur.2021.760813

**Published:** 2021-11-18

**Authors:** Julia Ferrari, Audrey Reynolds, Michael Knoflach, Marek Sykora

**Affiliations:** ^1^Department of Neurology, St John's Hospital, Vienna, Austria; ^2^Department of Neurology, St Vincent's University Hospital, Dublin, Ireland; ^3^Department of Neurology, Medical University of Innsbruck, Innsbruck, Austria; ^4^Medical Faculty, Sigmund Freud University Vienna, Vienna, Austria

**Keywords:** minor stroke, thrombolysis/thrombolytic agents, DAPT (dual antiplatelet therapy), very mild severity, rapidly improving stroke symptoms

## Abstract

Management of stroke with minor symptoms may represent a therapeutical dilemma as the hemorrhage risk of acute thrombolytic therapy may eventually outweigh the stroke severity. However, around 30% of patients presenting with minor stroke symptoms are ultimately left with disability. The objective of this review is to evaluate the current literature and evidence regarding the management of minor stroke, with a particular emphasis on the role of IV thrombolysis. Definition of minor stroke, pre-hospital recognition of minor stroke and stroke of unknown onset are discussed together with neuroimaging aspects and existing evidence for IV thrombolysis in minor strokes. Though current guidelines advise against the use of thrombolysis in those without clearly disabling symptoms due to a paucity of evidence, advanced imaging techniques may be able to identify those likely to benefit. Further research on this topic is ongoing.

## Introduction

A scenario known to every neurologist: a patient with acute onset mild stroke symptoms is admitted to the hospital. Imaging excludes an intracranial hemorrhage. Should intravenous thrombolysis be given? What are the risks and what are the benefits? It is frequently assumed that for those with mild stroke symptoms, risks of thrombolysis outweigh potential benefits. However, despite having “minor” symptoms, one-third of stroke patients were not functionally independent at 90 days when considered too mild to treat for intravenous thrombolysis (1–4). The purpose of this review is to provide an update on the acute treatment of patients with minor stroke with a special focus on intravenous thrombolysis.

## Definition of Stroke With Mild Symptoms

The definition of a stroke with mild symptoms or minor stroke (MS) is not standardized. Definitions are often based on the National Institutes of Health Stroke Scale (NIHSS) requiring a score ≤ 1 on every item (5) or utilize certain limits, mostly NIHSS ≤ 6 (6). Other definitions include whether symptoms are disabling or non-disabling, e.g., isolated aphasia or a severe distal paresis of the arm will give a low NIHSS score but are very disabling symptoms.

Further questions arise in differentiating minor stroke from a transient ischemic attack (TIA). In the acute phase, it is not possible to tell whether symptoms will persist or resolve spontaneously. The definition of a TIA from the American Heart and the American Stroke Association from 2002, “a transient episode of neurological dysfunction caused by focal brain, spinal cord, or retinal ischemia, without acute infarction” (7) implies the use of an advanced imaging method to differentiate between TIA and minor stroke. This definition will build the basis for the 11th International Classification of Diseases (8). A majority of TIAs are of short duration, and once neurological deficits persist longer than 60 min they resolve in <15% within 24 h (9). Furthermore, only 2% of patients that received placebo in the National Institute of Neurological Disorders and Stroke rt-PA Stroke Study were free of symptoms 24 h later (10).

Those with rapidly improving symptoms are usually excluded from receiving thrombolysis therapy. Rapidly improving symptoms are those which improve spontaneously after presentation, but the definition is ambiguous. However, their outcomes are not predictable, with 30% of those with minor stroke or rapidly improving symptoms not fully functionally independent at hospital discharge (1).

## Disabling or Non-Disabling Symptoms?

Determining whether symptoms are “disabling” or not is an important factor in the management of acute MS. A pooled metanalysis of nine trials could show that thrombolysis treatment resulted in a nearly 10% better chance of an excellent functional outcome after 3 months in patients with clearly disabling deficits such as aphasia or hemiparesis (11, 12).

For those with non-disabling symptoms, however, less evidence exists. Only one of these nine trials–the Third International Stroke Trial (IST-3) (13)–did not exclude patients with non-disabling symptoms.

IST-3 found evidence of benefit for thrombolysis for those presenting within 6 h of symptoms of stroke, however the benefit increased with increasing NIHSS and was less beneficial for those with minor stroke symptoms. Out of the 106 patients randomized with NIHSS ≤ 5, 60% showed a favorable outcome after 3 months.

Non-disabling symptoms include transient, fluctuating or persistent symptoms without unilateral motor weakness or language/speech disturbance (e.g., hemi-body sensory symptoms, monocular vision loss, binocular diplopia, hemifield vision loss, dysarthria, dysphagia, or ataxia). The PRISMS trial, a randomized controlled trial (RCT), showed that among patients with a low NIHSS and no disabling deficit, rtPA may not provide a benefit and might increase the risk of symptomatic intracranial hemorrhage (6). A clearly disabling deficit was operationally defined as a deficit that, if unchanged, would prevent the patient from performing basic activities of daily living (i.e., bathing, ambulating, toileting, hygiene, and eating) or returning to work. Judging how disabling a deficit will be in the future is challenging in the hyperacute stroke setting.

A further obstacle to thrombolysis treatment in minor stroke is that patients with minor stroke symptoms do not receive the priority of emergency medical services and in-hospital triage pathways leading to relevant time delays in onset-to-door and door-to-imaging times (14).

## Prehospital Recognition of Minor Stroke

The presentation of those with mild symptoms is frequently delayed compared to major stroke as it may not be recognized in the acute phase, leading to undertreatment. Public knowledge of stroke symptoms according to the FAST campaign is only about 70%, with the highest rate found in females and in the older and white population (15). Additionally, the mode of arrival at the hospital plays an important role. Patterns of emergency medical services pre-notification vary across countries. Data from a cohort study in New York showed that patients with minor stroke have longer door to needle times if the mode of arrival was without pre-notification (16).

The clinical significance of posterior circulation symptoms is often not recognized and, therefore, mostly remain undertreated in the acute phase. As in the NIHSS symptoms of the posterior circulation are underrepresented (e.g., vertigo, imbalance of gait), strokes in this territory are more likely to be defined as “minor” if a cut-off NIHSS score is used.

## Wake up Stroke and Stroke of Unknown Onset

Those who wake up with stroke were traditionally excluded from revascularization therapies, due to unknown time of onset. Due to circadian rhythms there is diurnal variation in stroke onset, with a higher number occurring in the morning, which may be related to a surge in blood pressure (17). This suggests that the stroke may have occurred shortly before awakening, though the true time of onset is unknown. Modern imaging technologies, such as MRI DWI and FLAIR mismatch and or perfusion imaging, can help identify those who may benefit from thrombolysis or thrombectomy (18). The WAKE-UP trial showed that those with strokes evident from sleep with favorable MRI findings (DWI and FLAIR mismatch) who were treated with IV alteplase had significantly better functional outcomes, though more intracranial hemorrhages, than placebo at 90 days (19). The WAKE-UP trial included patients with all types of stroke severity, but the median NIHSS was of mild to moderate severity (median NIHSS 6, interquartile range 4–9). Analysis of patients with minor stroke has not been reported so far. Penumbral pattern identified using perfusion imaging is another recent radiological paradigm to identify those to benefit from reperfusion in the absence of onset time knowledge. Trials including ECASS4, EPITHET and EXTEND proved positively this concept for wake-up strokes and extended time window (4.5–9 h) thrombolysis (20).

Thrombolysis for wake-up stroke with minor symptoms has not been specifically studied. As mentioned previously, many stroke centers do not perform advanced imaging in those with NIHSS ≤ 6, and may be missing those with mismatch deficits or large vessel occlusions who could potentially benefit from thrombolysis. See also illustrative patient case in **Figure 2**.

## Current Evidence of use of Thrombolytic Agents in Patients With Minor Stroke

Current guidelines and recommendations state that for patients with acute minor disabling ischemic stroke of <4.5 h duration, intravenous thrombolysis with recombinant alteplase is recommended/ may be reasonable (21, 22). RCTs and observational studies addressing this topic so far showed promising results with a good functional outcome and a low complication rate (Table 1).

**Table 1:** Randomized controlled trials and observational trials on thrombolysis in minor stroke.

**Reference**	**Patient group**	**Study type**	**Inter-vention**	**Outcome**	**Key results**	**sICH**	**Mortality**
IST 3 Sandercock et al. (13); Khatri et al. (23)	NIHSS ≤ 5 within 3 h of onset *n* = 106	International, multicentre, randomized, controlled	rtPA	Alive and independent at 6 month Oxford handicap scale (OHS) 0–2 and favorable outcome after 6 month (OHS 0–1)	Alive & Independent (OHS 0–2): 84 vs. 65%, aOR 3.3, 95% CI 1.2, 8.8 ∙ Favorable outcome (OHS 0–1): 60 vs. 51%, aOR 1.9, 95% CI 0.8, 4.4	0%	0%
Emberson et al. (11)	NIHSS <5 *n* = 666	Metaanalysis	rtPA	mRS 0–1 at 3 months	OR 1.48, CI: 1.07–2.06, favoring rtPA	0,9%	NA
TEMPO 1, Coutts et al. (24)	NIHSS <5 *n* = 50	Phase 2, randomized, open label	0.1 mg/kg TNK 0.2 mg/kg TNK	Rate of expected serious adverse events.	No serious drug-related adverse events in 0.1 mg/kg group. In the 0.25 mg/ kg group, 1 sICH	0.25 mg/kg group: 4%	0.25 mg/kg group: 4%
PRISMS, Khatri et al. 2018 (6)	non-disabling NIHSS ≤ 5 *n* = 313	Phase 3b, randomized, blinded	rtPA	mRS 0–1 at 3 months	78.2% in the rtPA group vs. 81.5% in the aspirin group (adjusted risk difference, −1.1%; 95% CI, −9.4% to 7.3%)	3.2%	0.6%
Khatri et al. (25)	*n* = 38, NIHSS ≤ 5	Retrospective analysis from the NINDS trial	rtPA	mRS of 0–1 at 3 months	78.6% (CI 63.2–89.7%) of rtPA cases vs. 81.3% (CI 54.4–96.0%) of the placebo cases	2,4%	1 patient died
Sykora et al.2021 (26)	*n* = 703, NIHSS 0–1	Retrospective	rtPA	mRS of 0-1 at 3 months	75.5% rtPA vs. 80.8% non-rt-PA group, adjusted OR 0.57, CI 0.4–0.81	1.4%	4.7%
Huisa et al. (27)	*n* = 133, NIHSS ≤ 5	Retrospective	rtPA	mRS of 0–1 at 3 months	57.6% of the rtPA group and 68.9% of the untreated group (OR 0.93, CI 0.39–2.2)	5%	5,1%
Urra et al. (28)	*n* = 203, NIHSS ≤ 5	Prospective observational	rtPA	mRS of 0–1 at 3 months	167 (82%) patients had excellent outcome; thrombolysis was associated with a greater proportion of patients who shifted down on the modified Rankin Scale score at 3 months (OR 2.66; CI 1.49–4.74, *p* = 0.001).	0%	1,7%
Greisenegger et al. (29)	*n* = 890, NIHSS ≤ 5	Retrospective	rtPA	mRS of 0–1 at 3 months	OR 1.49; CI 1.17–1.89; *P* <0.001 favoring rtPA cases	2,5%	N/A

*NIHSS, National Institute of Health Stroke Scale; mRS, modified Rankin scale; rtPA, recombinant tissue plasminogen activator; sICH, symptomatic intracranial hemorrhage*.

For patients with acute minor non-disabling ischemic stroke of <4.5 h duration, no intravenous thrombolysis is recommended. One exception may be patients with non-disabling symptoms and a large vessel occlusion. However, many acute stroke centers do not perform angiography for those with NIHSS <6 as part of their internal protocol, and many centers do not have access to advanced imaging such as CT perfusion. Therefore, an unknown proportion of stroke with minor symptoms who have large vessel occlusions amenable to intervention are being missed. TEMPO 1, a case series of 50 patients with mild symptoms and intracranial vessel occlusion, which showed that administration of tenecteplase-tissue-type plasminogen activator in minor stroke with intracranial occlusion is feasible and safe (24). Wang et al. found that intravenous thrombolysis benefits though with mild stroke symptoms (NIHSS ≤ 5) and large artery atherosclerosis, though not those who had a tandem proximal intracranial occlusion and cervical internal artery lesion (complete occlusion or severe stenosis ≥ 90%) (30). They found that LAA-type patients (as defined by TOAST criteria) had significantly favorable outcomes after treatment with thrombolysis compared to untreated patients, however no such benefits were observed in other stroke subtypes, such as cardioembolic, small vessel occlusion and undetermined. This suggests that CT or MR angiography might be helpful to choose patients for thrombolysis that present with stroke with minor symptoms.

## Alteplase or Tenecteplase in Patients With Minor Stroke

In recent years, the recombinant plasminogen activator tenecteplase is increasingly competing with the gold standard alteplase. The first publication of the EXTEND IA TNK study showed that higher perfusion rates and better clinical results can be achieved with tenecteplase in the 0.25 mg/kg dose than with alteplase in patients with an acute ischemic stroke (31). Tenecteplase was used as so-called bridging thrombolysis in the 4.5 h time window until the mechanical thrombectomy was performed. In addition, tenecteplase has advantages in handling, as it can be administered as single intravenous bolus and does not require a continuous infusion over 1 h, as alteplase does. The results of the EXTEND TNK study prompted the authors of the US guideline and the European Stroke Organization (ESO) to include tenecteplase in their recommendation as an alternative fibrinolytic (AHA/ASA Class IIb recommendation), although the AHA/ASA recommendation can also be considered to the 0.4 mg/kg dose for patients with less severe neurological impairments and if there are no large vessel occlusions (Level of Evidence: IIb) (22).

The second part of the EXTEND TNK study was recently published (32) which evaluated different doses of tenecteplase. The higher dose of tenecteplase (0.4 mg/kg) did not have any disadvantages in terms of safety: there were 16 and 22 death in the high and low dose groups, respectively. Symptomatic intracerebral hemorrhages 36 h after thrombolysis were numerically more frequent in the high dose group (7 vs. 2 patients), but four bleeding events in this group were associated with wire perforations during the endovascular procedure and were therefore not attributable to thrombolysis directly. The authors of the study report that the latter results are in contrast to an earlier study with the 0.40 mg/kg dose that was terminated prematurely for safety reasons, as some patients developed symptomatic intracranial hemorrhage. As a limitation, Campbell and colleagues point out that the study may not have been powered to reveal differences in efficacy. There was no restriction on clinical severity using NIHSS scores in these trials, but showed that probably a higher perfusion rate can be achieved with tenecteplase in patients with vessel occlusions. TEMPO 2 is an ongoing multicentre prospective randomized open label blinded-endpoint (PROBE) controlled trial of thrombolysis with low dose TEnecteplase vs. standard of care in Minor ischemic stroke with Proven acute symptomatic Occlusion (33). The hypothesis is that patients with mild (NIHSS < = 5) or even non-disabling symptoms due to identifiable vessel occlusion will benefit from IVT as compared to standard antiplatelet therapy. Results are expected in 2024. In summary, currently no evidence exists that tenecteplase should be preferred to alteplase in acute treatment of minor stroke patients, though further research is ongoing.

## Time Trends of Using Intravenous Thrombolysis

In Austria, rates of rtPA treatment in patients with very mild symptoms (NIHSS 0-1) raised from 0.8% in 2006 to 3.5% in 2018 and for patients with a NIHSS 2–3 from 2.2% in 2006 to 17.2% in 2018 (34). Another large registry from 66 hospitals in Puerto Rico and Florida reported a substantial increase in thrombolysis rates of patients with minor stroke presenting within 4 h of stroke onset from 10% in 2010 to 25% in 2015 (14). The Get With the Guidelines Stroke database, which collects information from 1,783 hospitals across the United States, showed that the use of thrombolysis has increased from 45% in 2003 to 2005 to 82% in 2010 to 2011 (35). These substantial increases rtPA use in ischemic stroke patients with mild symptoms in different parts of the world document an increasing confidence in using this treatment according to the guidelines, which are regularly updated with regard to the minor stroke patient group.

Data from a prospective stroke thrombolysis registry in France (36) showed that a high rate (77%) of excellent outcome (3 month-modified Rankin Scale score ≤ 1) was observed in 1,035 minor stroke patients receiving thrombolysis. No symptomatic intracerebral hemorrhage occurred and the rate of any hemorrhagic transformation was 5%.

## Patients With Very Mild Symptoms

A recent analysis from a large nationwide stroke registry in Austria shows that in patients with very mild symptoms (NIHSS 0–1), treatment with intravenous thrombolysis did not increase the likelihood of an excellent outcome as compared with those managed conservatively. On the contrary, those receiving IVT were more likely to suffer early neurological deterioration (adjusted OR 8.84, CI 6.61–11.83), symptomatic intracranial hemorrhage (adjusted OR 9.32, CI 4.53–19.15) and lower rate of excellent outcome (mRS 0–1) at 3 months (adjusted OR 0.67, CI 0.5–0.9). Proposed explanations for this phenomenon may include large vessel occlusion, thrombus migration, reperfusion injury, or re-embolization (26). Indeed, up to a third of patients of patients with initially mild symptoms may harbor a large vessel occlusion which may not respond well to IVT alone and may lead to secondary deterioration (37).

## Dual Antiplatelet Therapy

If thrombolysis treatment is contraindicated or clinical assessment does preclude its use, current guidelines recommend dual antiplatelet therapy with Aspirin and Clopidogrel or Aspirin and Ticagrelor for a short time in patients with a minor stroke (NIHSS score ≤ 3) or high-risk TIA (ABCD2 score ≥ 4) (38). This treatment does not aim at vessel recanalization or rapid improvement of stroke symptoms, but rather to reduce early stroke recurrence.

The CHANCE study used a 300-mg clopidogrel (then 75 mg daily) and an aspirin loading dose of 75 to 300 mg followed by 75 mg daily within the first 24 h after TIA or minor stroke for a duration of 21 days (39). In the POINT trial 600-mg clopidogrel loading dose (then 75 mg daily) and an aspirin regimen of 50 to 325 mg daily started within in the first 12 h after TIA or minor stroke for up to 90 days was used (40). Additional analysis of the POINT trial could show that the effect of avoiding a recurrent stroke is primarily seen in the first 21 days, so that the recommendation is to treat these patients with a DAPT with loading doses immediately after TIA or minor stroke for no longer than 21 days. In both the CHANCE and POINT trials the benefits clearly outweighed the risk of bleeding.

In the THALES trial the use of ticagrelor (180-mg loading dose, then 90 mg twice daily) plus aspirin (300- to 325- mg loading does, then 75–100 mg daily) for 30 days was shown to be slightly superior to aspirin alone in preventing recurrent stroke with a significant increase in bleeding (41). In this study the preventive effect of the DAPT outweighed the risk of bleeding.

RCT's regarding the comparison of thrombolysis vs. DAPT in acute minor stroke patients are lacking. There is only one exploratory comparative analysis (42) showing a weak trend among intravenous thrombolysis, DAPT and Aspirin but not a significant difference in 90 day functional outcome in patients with minor stroke.

## Discussion

Traditionally those presenting acutely with stroke with minor symptoms have been excluded from thrombolysis due to concerns that risks of hemorrhage would outweigh the benefits. However, as we have discussed above those presenting lower NIHSS scores may still experience long term disability. Currently it is challenging to judge what patients are likely to benefit most from thrombolysis (with or without thrombectomy) treatment with the lowest risk of bleeding complications.

**Figure 1:**
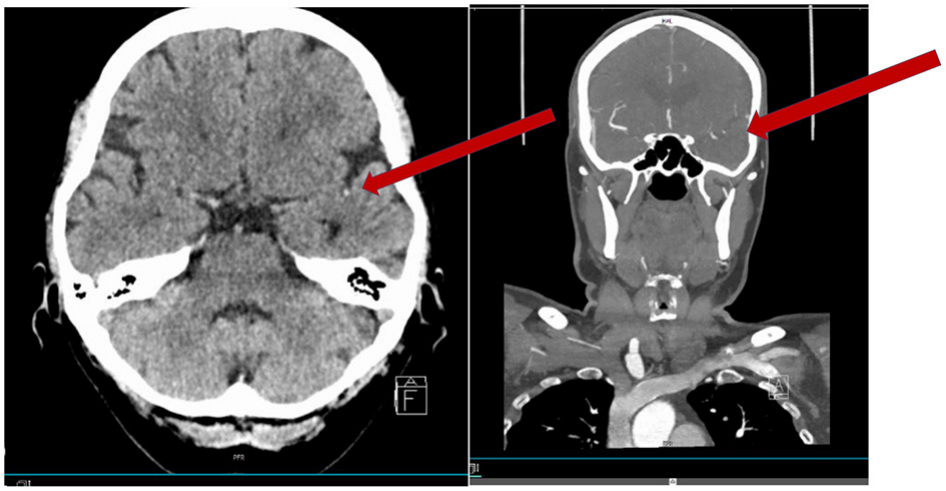
Fifty-nine-year-old woman with an acute onset of aphasia (NIHSS 1 disabling). CT scan showing a hyperdense vessel sign in M2 (arrow). CTA showing the occlusion in M2 (short in length).

**Figure 2:**
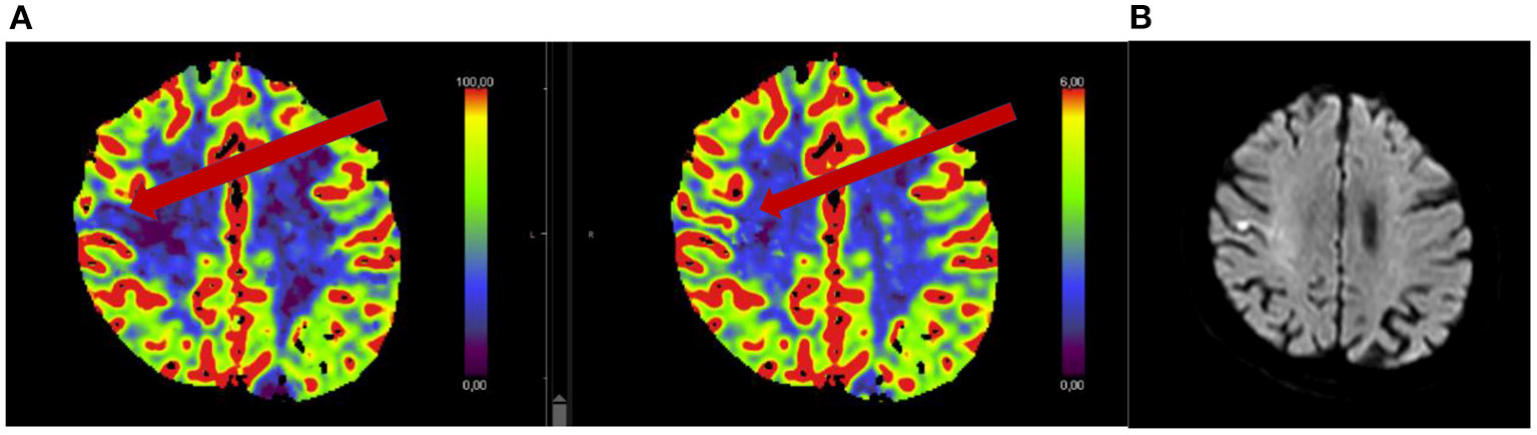
Seventy-five year old woman with acute onset of mild left hemi-body weakness and sensory loss (NIHSS 3). Patient received intravenous thrombolysis and showed clinical improvement. Acute CT perfusion showed a mismatch between normal cerebral blood volume **(B)** and reduced cerebral blood flow **(A)**. Follow-up MRI performed 24 h after thrombolysis showed a small cortical ischemia.

Patients with vessel occlusions (like patients in Figures 1, 2) are likely to benefit from recanalizing treatments rather than (intensified) secondary prophylaxis with antiplatelets. Yet, in a majority of centers acute stroke treatment protocols do exclude patients with a low NIHSS from advanced imaging like MR/CT-angiography and perfusion. Therefore, the decision to initiate and organize these imaging modalities leads to time delays making thrombolysis less safe and efficient. Indeed, a recent study showed significant increased detection of LVO and increased frequencies of performed MTs after an in-house protocol change excluding the NIHSS criterion (43). Therefore, we would advocate that all patients presenting with stroke symptoms, including minor stroke symptoms, have angiography (typically CT) as part of their acute work up.

There is some data which suggests that minor strokes of certain etiologies–e.g., like strokes due to large artery atherosclerosis (without large vessel occlusion) (30)–may benefit more from thrombolytic treatment.

**Figure 3:**
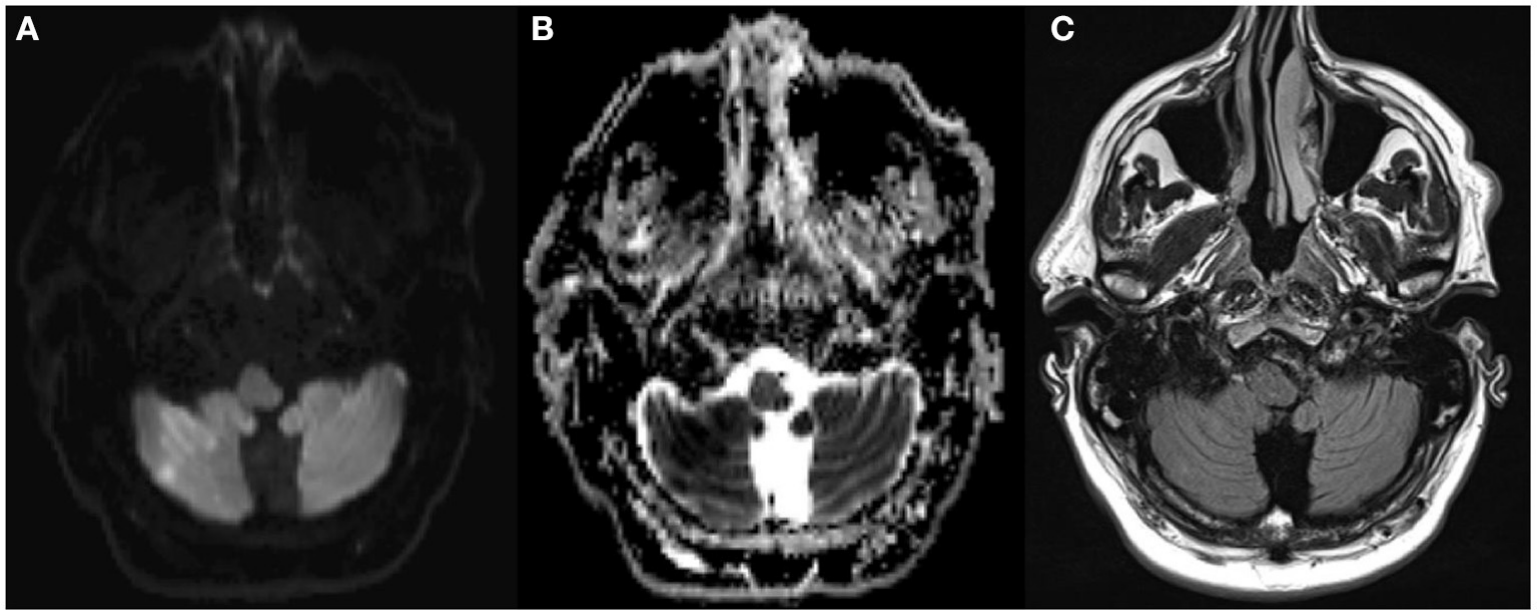
Fifty-five year old man with acute onset of mild dysathria and ataxia of the trunk, unable to walk (NIHSS 1). MRI showed an acute infarction [DWI positive **(A)**, ADC negative **(B)**, FLAIR negative **(C)**] in the territory of the occluded right posterior inferior cerebellar artery. The patient was treated with rtPA 3 h and 46 min after stroke onset.

Finally, the relevance of a neurological deficit can be extremely hard to judge in the acute stroke setting. Symptoms such as neglect, extinction, and cognitive deficits can be frequently under recognized in the emergency room. In particular, posterior circulation strokes tend to be misclassified as “minor stroke” (illustrative patient example Figure 3).

The NIHSS has limitations with respect to its use when comparing the neurologic severity of a posterior circulation stroke and anterior circulation stroke (44). A patient with an acute ischemic stroke in the posterior circulation might have a comparably low score like patients with an acute ischemic stroke in the anterior circulation but be bedridden due to severe ataxia and/or vertigo, underlining that patients with a posterior circulation stroke need the same diagnostic and therapeutic measures (e.g., iv thrombolysis) like patients with an anterior circulation stroke (45).

## Conclusion

Patients with a minor stroke are by no means to be classified as benign and may result in lasting significant neurological deficits. Even though the use of IV thrombolysis in this setting has substantially increased world-wild, current guidelines still recommend the administration of thrombolysis only to minor stroke patients with clear disabling symptoms, due to lack of convincing data from large randomized-controlled trials. Advanced imaging might help to better estimate the risk-benefit ratio of thrombolysis treatment in acute ischemic stroke with minor symptoms. Further results of ongoing trials on this topic are expected shortly.

## Author Contributions

All authors contributed to the writing of the manuscript, literature search and provided patients cases. All authors read and approved the final version of the manuscript.

## Conflict of Interest

The authors declare that the research was conducted in the absence of any commercial or financial relationships that could be construed as a potential conflict of interest.

## Publisher's Note

All claims expressed in this article are solely those of the authors and do not necessarily represent those of their affiliated organizations, or those of the publisher, the editors and the reviewers. Any product that may be evaluated in this article, or claim that may be made by its manufacturer, is not guaranteed or endorsed by the publisher.
